# Unravelling the link between circadian clock genes and brain tumors: From pathological disruptions to potential therapeutic interventions

**DOI:** 10.3389/fphar.2025.1617713

**Published:** 2025-05-27

**Authors:** Amirah Albaqami

**Affiliations:** Department of Clinical Laboratory Sciences, Turabah University College, Taif University, Taif, Saudi Arabia

**Keywords:** circadian clock genes, brain tumors, glioblastoma multiforme, pathological disruptions, pharmacological interventions

## Abstract

The circadian clock is present in nearly all tissues (including glial cells), which play crucial roles in brain functions and development. Circadian clock genes (CCGs) are responsible for regulating numerous cancer-associated biological mechanisms, including the regulation of cell cycle genes, apoptosis, and cell proliferation. A range of studies have already confirmed the potential link between brain tumors and CCGs, including *Bmal1, Clock, Period 1, Period 2, Period 3, Cry1, Cry2, retinoid-related orphan receptor-α (ROR-α).* Growing evidence regarding gliomas including glioblastoma multiforme (GBM) indicates the significance of modulation of CCG in cancer biology. Various studies have already revealed how tumor cells can disrupt CCGs to safeguard their survival. It has also recently been demonstrated in the case of gliomas (especially GBM) that CCGs should be targeted for the development of novel therapies or to ameliorate the current treatments that impair and abolish tumor growth. Multiple pharmacological modulators have been reported as effective in regulating CCGs in brain tumors, such as temozolomide, inhibitors of casein kinase 1 and casein kinase 2, curcumin, norepinephrine, melatonin, REV-ERB agonists, agonists of the retinoic acid-related orphan receptor, cryptochrome protein stabilizers, and 1A-116. In this review, an overview of brain tumors, the genetics of circadian clock, and the link between pathological disruptions of the CCGs and brain tumor development have been discussed. In addition, potential pharmacological interventions to modulate CCGs in brain tumors have also been reviewed.

## 1 Introduction

The suprachiasmatic nucleus (SCN) in the hypothalamus is known as the central circadian pacemaker, which regulates many biological mechanisms in the body. Circadian clock (CC) genes (CCGs) are responsible for regulating numerous cancer-associated biological mechanisms, including the regulation of cell cycle genes, apoptosis, and cell proliferation (Yu and Weaver, 2011). Inherited mutations or environmental disruption of CCGs might negatively influence cellular activities, which can result in cancer. A range of studies have already confirmed the link between variants in CCGs and risks of non-Hodgkin lymphoma, prostate cancer, and breast cancer (Hoffman et al., 2010; Yi et al., 2010; Lin et al., 2011; Zhao et al., 2012; Zhou et al., 2012). Consistently, a potential link between CCGs and brain tumors (BTs) has also been confirmed. It was observed that as compared to adjacent non-glioma cells, period CC (*Per*) genes including *Per1* and *Per2* were found to be underexpressed in glioma cells (Xia et al., 2010). A similar trend has also been observed in the case of other CCGs, including cryptochrome (CRY) genes (*Cry1* and *Cry2*) (Madden et al., 2014).

BTs involve a diverse group of central nervous system (CNS) neoplasms. Depending on the pathological diagnosis, around 100 different types of BTs have been recognised. Depending on whether these tumours originate in the brain or merely spread to the brain from other parts of the body, they can generally be grouped into primary or secondary BTs. Malignant gliomas represent almost half of all primary BTs, while astrocytomas represent over three-quarters of all gliomas. In addition, malignant gliomas represent 28% of all BTs and 2% of all cancer types. Global age-adjusted occurrence of all gliomas ranges from 4.67 to 5.73 per 100,000 people (Ostrom et al., 2014; Weller et al., 2015; Davis, 2018; Louis et al., 2021). Glial tumours occur from glial precursor cells, oligodendrocytes or astrocytes (Liu et al., 2011; Lee et al., 2018). Gliomas can be classified as grade I (least aggressive) to grade IV (most aggressive), depending on the molecular markers and diverse histological features including intratumoral necrosis, vascular proliferation, nuclear polymorphisms, and cellular heterogeneity.

Increasing evidence suggests that the deregulation of CCGs is associated with gliomagenesis. CCG expression levels in the high-grade glioma were substantially higher as compared to non-gliomas or low-grade gliomas. Moreover, disrupted CC can lead to impaired stemness of glioma stem cells (GSCs) in glioblastoma multiforme (GBM) (Huang et al., 2019). In a study, Petkovic et al. characterized glioma-associated dysregulation of CC and CCGs. These researchers demonstrated that an increased level of dysregulation in gene expression is present in GBM as compared to low-grade glioma, which further suggests the link between the differentially expressed clock-controlled genes and survival of the patients in case of both GBM and low-grade glioma (Petkovic et al., 2023a). Patients with BTs might experience disturbances in their CC, which can be manifested by disruptions in activity patterns, abnormal mental states, and sleep disorders. Therefore, assessment of the link between CCGs and BTs can help in understanding how CC disruptions can affect the quality of life of patients with BTs and progression of the disease (Hou et al., 2024). In a study, Hou et al. (2024) explored the link between core CCGs and multiple characteristics of BT pathogenesis. In order to carry out comprehensive analyses, they used various datasets including mutation, methylation, gene expression, and clinical data from patients with brain tumor. The researchers studied the impact of CCGs in the development of lower grade glioma and observed that certain genes including *RORβ*, *NPAS2,* and *CRY1* were linked with elevated or reduced risk of lower grade glioma (Hou et al., 2024). The researchers also assessed the link between CCGs and immune cell infiltration, which revealed a positive relationship with infiltration of B cells and CD8+T cells and a negative relationship with macrophage infiltration. Moreover, the researchers detected the main mutated CCGs, including *Clock, BMAL1, BMAL2*, and *Per2*, and their possible interaction with various other CNS-related genes. It was concluded that CCGs have a significant contribution in immune responses as well as tumorigenesis in patients with lower grade glioma, which also requires more investigations.

CC orchestrates various physiological mechanisms by modulating cell functions and signalling pathways. This role further extends to the differentiation, behaviours, and functions of immune cells associated with innate and adaptive immunity systems (Wang Q. et al., 2022; Cermakian and Labrecque, 2023). There is a growing number of studies focusing on the CC-mediated regulation of transcriptional processes that determine gene expression in key signalling pathways, including Janus kinase/signal transducer and activator of transcription, mitogen-activated protein kinase (MAPK), and nuclear factor-κB (Dong Q. et al., 2019; Habbel et al., 2020). Disrupted circadian rhythms can worsen the malfunctioning of these regulatory mechanisms, potentially resulting in uncontrollable inflammatory responses and diseases (Lin et al., 2024). It is now well-known that circadian rhythms have a significant influence on numerous parameters in the immune system including the cytokine levels and the number of circulating hematopoietic cells (Cermakian et al., 2013; Nakao, 2014). Various studies have already demonstrated that CC plays an important role in the regulation of cytokines (Bechtold et al., 2010; Arjona et al., 2012; Scheiermann et al., 2013). In a study, Keller et al. (2009) observed that macrophages derived from the mouse peritoneal cavity, lymph nodes, and spleen contain intrinsic CC that operate independently as well as other acquired and innate immune cells. The researchers also reported that macrophages derived from mouse spleen induced with LPS at different time points showed circadian rhythms in the secretions of various cytokines including interleukin-6 and tumor necrosis factor alpha (TNF-α), which indicates that macrophage-intrinsic CC might regulate these oscillations (Keller et al., 2009).

It has been observed that CD8^+^ T cells also express CCGs and their counts show 24-h rhythms in the blood and in secondary lymphoid organs, which was found to rely on the CC present in these cells and on hormonal rhythms. Furthermore, the extent of responses mediated by CD8^+^ T cells to antigen presentation can vary depending on the time of day, a circadian rhythm reliant on the CD8^+^ T cell clock (Cermakian and Labrecque, 2023). Circadian control of immune modulation has a significant contribution in tumor immunosurveillance and host defence (Fortin et al., 2024). In a study, Wu et al. (2019a) reported that immune checkpoint expression and immunoregulatory mechanisms can be affected by the disturbance in circadian rhythmicity in tumour-resident cells to benefit the tumor.

Crespo et al. (2012) observed a differential pattern of CCG expression in glioma cells in comparison with their paired neighbouring normal brain tissues, which suggests an asynchrony amongst the CCs. An altered chromosomal number was also detected by the researchers utilizing single nucleotide polymorphism array. In addition, the amplification of chromosomal segment 4q12 was identified, where the mammalian *CCG* is situated. The altered copy number on the DNA level had an impact on the mRNA levels, which further indicates an important link with the disease pathogenesis (Crespo et al., 2012). In GSCs, it has been observed that downregulation of the *Bmal1* gene stimulated apoptosis as well as cell cycle arrest (Dong Z. et al., 2019). Epithelial-mesenchymal transition (EMT) is a process that mediates GSCs populations, invasiveness, and tumor cell metastasis (Hakami et al., 2024a). The targeted phase activation in the core CCG *Per2* might serve as a potential target for therapies that might inhibit EMT, limit tumor metastasis, and minimize GSCs (De et al., 2020). The expression of the *Per2* gene was found to be enriched within C6 glioma tumor spheres but not in monolayer cell culture, suggesting that cell interactions or tumor microenvironment (TME) permit circadian timing (Wang and Chen, 2022). The GBM heterogeneity may be partly bestowed by the phenotypic plasticity indigenous to cancer stem cells mediating adaptability needed for tumor growth (Safa et al., 2015).

The most malignant glioma (grade IV) is known as GBM, which is the most aggressive type of BT and patients with GBM have an average survival of 15 months despite multimodal therapies including chemotherapy, radiation therapy, and surgery (Weller et al., 2015; Ostrom et al., 2019a; Petkovic et al., 2023b). Numerous novel therapies have been developed to treat GBM, however after diagnosis still less than 5% of patients with GBM survive for 5 years (Davis, 2016). In recent times, several researchers suggested improving the delivery of anticancer drugs via timing it to the daily rhythms of the patients (Dakup et al., 2018). CCs primarily organize the behavior and physiology of humans via producing daily rhythms in various physiological mechanisms, including endocrine systems, digestive and cardiovascular mechanisms, body temperature cycles, locomotor function, behaviour, sleep/wake cycles, and immune and metabolic activities with an intrinsic 24-h period oscillation. This approach has recently been utilized in the treatment of BTs owing to a differential response to bortezomib, a proteasome inhibitor, in a murine model. On the other hand, temozolomide (TMZ) chronotherapy in murine and human GBM cells in culture was found to be reliant on CCG expression.

CC plays an important role in regulating various metabolic pathways including glycolysis. Thus, disruption of circadian rhythm is linked with metabolic imbalance (Yoo et al., 2020). Indeed, glucose homeostasis is regulated via the CC present in the SCN and peripheral clocks present in the white adipose tissue, pancreas, muscle, and liver. Glucose present in blood is mainly obtained through diet during the active phase and predominantly from endogenous glucose generation in the liver during the resting phase. The uptake of glucose shows a 24-h rhythm, along with the highest level at the beginning of the active phase and the lowest level at the beginning of the passive phase (Kalsbeek et al., 2014; Zlacká and Zeman, 2021a). Chronodisrupted individuals exhibit a disturbed rhythm in plasma glucose as well as insulin levels. In addition, genetic studies revealed that there is a link between PER2 and CRY and blood glucose level (Gachon et al., 2017).

Mitochondria has a significant contribution in oxidative phosphorylation. It has been observed in rat hepatocytes that the shape and volume of mitochondria can oscillate under dark and light conditions. Hepatocytes derived from mice at different times during the day showed increased levels of respiration during the dark as compared to the light phase in the presence of pyruvate (Zlacká and Zeman, 2021b). In a study, Schmitt et al. (2018) reported that CC-regulated oxidative phosphorylation was reliant on dynamin-related protein 1 (DRP1). DRP1 activities are controlled by phosphorylation at serine residue 637 (Ser637) and following inactivation or activation. Phosphorylation of DRP1 at Ser637 exhibits 24-h rhythms along with the peak level at CT12, which is beginning of the subjective night (Schmitt et al., 2018). On the other hand, oxidative stress is linked with the pathogenesis of various diseases and the interaction between CCs and oxidative stress is evident, where disrupted circadian rhythms can modify redox homeostasis resulting in oxidative stress and increased generation of reactive oxygen and nitrogen species might trigger circadian oscillations (Wilking et al., 2013; McClean and Davison, 2022).

Circadian rhythm can also affect the pharmacodynamics and pharmacokinetics of anticancer therapies. Knowledge regarding the processes underlying chemoresistance can facilitate identification of a group of patients who might benefit from chemotherapy and circumvent overtreatment (Brahimi-Horn et al., 2012). *In a study,* Fang et al. (2015) *reported that degradation of CCG CRY2 Is associated with the chemoresistance of colorectal cancer. In another study,* Xu et al. (2018) revealed that CCG *CLOCK* is strongly linked with chemo-resistance of ovarian cancer cells. Increased expression of *CLOCK* endowed resistance of ovarian cancer cells to cisplatin treatment. It was observed that CLOCK-induced increased level of drug resistance genes (such as P-glycoprotein (*P-gp*)), autophagy, and ATP binding cassette subfamily C member 2 may facilitate the aforementioned process (Sun et al., 2017). In this review, an overview of BTs, the genetics of the circadian clock, and the connection between pathological disruptions of the CCGs and BT development have been discussed. Moreover, potential pharmacological interventions to modulate CCGs in BTs have also been reviewed.

## 2 Brain tumors and circadian clock

BTs including both primary and secondary tumours are graded from I to IV according to World Health Organization (WHO) (Kheirollahi et al., 2015). Grade I tumors exhibit a slow proliferative rate, which can be treated by surgical resection. Grade II tumours are low-grade gliomas that show less proliferation activity; however they show greater infiltration activities and can even progress to higher-grade tumors. Patients with grade II tumors have an overall over 5 years survival rate. Surgery can be carried out to treat grade II tumors, however these tumors frequently recur more aggressively (Wirsching and Weller, 2017). On the other hand, grade III tumors show an increased rate of infiltration and high proliferative activity. In addition to this, Grade III tumors are histologically malignant and patients have an overall 2–3 years survival rate, which can be treated with chemotherapy and/or adjuvant radiation therapy. The most vigorous proliferation activities are exhibited by life-threatening grade IV tumors, which can even invade adjacent tissues. Grade IV tumors show abrupt mitotic activity, which needs more aggressive therapies containing chemotherapy as well as adjuvant radiation therapy (Louis et al., 2016). Gliomas and neuroepithelial tumors are the most common form of primary BTs, whereas meningiomas are the most common secondary BTs (Baldi and Loiseau, 2012). Both males and females are equally prone towards BTs, however females tend to develop meningiomas more than males (Sun et al., 2015; Rasheed et al., 2021). In the USA, primary BTs are one of the top 10 causes of tumor-associated deaths as per the American Cancer Society. Around 13,000 BT-associated deaths are reported every year in the USA. In addition, 1 in 1,300 children aged below 20 is diagnosed with BT (Baldi and Loiseau, 2012). Various factors (for example-genetic risk factors, exposure to ionizing radiation and pesticides) are responsible for BTs and currently used therapies in the treatment of BTs have poor therapeutic efficacy and serious side effects (Ostrom et al., 2019b). Furthermore, the blood-brain barrier (BBB) poses numerous challenges in the delivery of anticancer drugs because of the tight junctions of the BBB, which severely restrict the delivery of drugs into the brain at the targeted tumor sites (Rasheed et al., 2021; Kaur et al., 2023).

Circadian disruption has been identified by WHO as a possible carcinogen (Munteanu et al., 2024). CC alterations have also been associated with elevated risk of various cancers including lung, ovary, pancreas, liver, colon, breast, and prostate cancers. In addition, a deficiency of circadian control is associated with insufficient efficacy of anticancer therapies and early mortality of cancer patients (de Assis and Oster, 2021). Interestingly, cancer, diabetes, obesity, mood disorders, and sleep are associated with the alterations in the CC caused by not getting enough sleep, eating at night, or even chronic jet lag (de Assis and Oster, 2021). Functions and expressions of various oncogenes and tumor suppressors in both tumor tissues and host are substantially changed via CC disruptions caused by environmental and genetic factors. Moreover, CC disturbances can reposition the host immune and metabolism systems, which can mediate an immunosuppressive TME in various types of cancers (Lee, 2021). Recently, a direct link between the core CC and apoptosis has been demonstrated. According to the clock status and cellular context, circadian factors can both restrict and mediate apoptosis, as was observed with the regulation of cell cycle. In terms of mediating cell death, PER1 and cryptochrome (a photoreceptor associated with the CC) can influence the extrinsic TNF-α-dependent pathway and the intrinsic apoptotic pathway, respectively (Shafi and Knudsen, 2019). Moreover, at least 14 core CCGs form several chain feedback loops that mediate the CC along with its intrinsic circadian rhythmicity. Numerous studies have already demonstrated a strong link between the risk of various cancer types CCG dysfunctions caused by epigenetic modification, deletions, single nucleotide polymorphisms, and deregulation to the impact that CCGs have in the onset and metastasis of cancer (Mocellin et al., 2018; Liu et al., 2022; Zhang et al., 2024).

## 3 Genetics of circadian clock

Research on *Drosophila* mutants with aberrant behavioral rhythms led to the discovery of CCG, *period (per)*. Indeed, these investigations laid the basis for knowledge regarding the molecular foundation of the CC, as the *Per* gene regulates the PER protein and CC, where PER protein itself controls the expression of *per* gene. Interestingly, the first known mammalian central CCG, *Clock, was revealed by extending the investigations in Drosophila, which was further evaluated in* forward-genetic analysis in mice with aberrant CCs. The PER protein in *Drosophila* was found to have common features with the CLOCK protein in mice, for example, a PAS domain (for Sim, ARNT, and Per). Nonetheless, the CLOCK and BMAL1 work together to regulate CCs, they also contain bHLH domains that facilitate the binding of DNAs directly with the regulatory elements (E-boxes) on rhythmic genes to regulate their transcription (Cox and Takahashi, 2019). The main targets of CLOCK:BMAL1 involve various other core CCGs that encode the CRY proteins (encoded by *Cry1* and *Cry2* genes) and PER proteins (encoded by *Per1, Per2*, and *Per3 genes).* These negative controllers heterodimerize then translocate into the nucleus, where they inhibit their own gene transcription through direct interaction with the CLOCK:BMAL1 (Michael et al., 2017; Rosensweig et al., 2018). Furthermore, the mRNA expressions of *Cry1/2* and *Per1/2/3* are controlled through various processes (Kojima et al., 2011). For example, degradations of CRY and PER proteins are controlled via the F-box proteins (including FBXL21 and FBXL3), casein kinase 1 epsilon (CK1ε) as well as delta (CK1δ), serine/threonine kinases, and various other proteins (Hirano et al., 2013; Yoo et al., 2013; Narasimamurthy et al., 2018).

**FIGURE 1:**
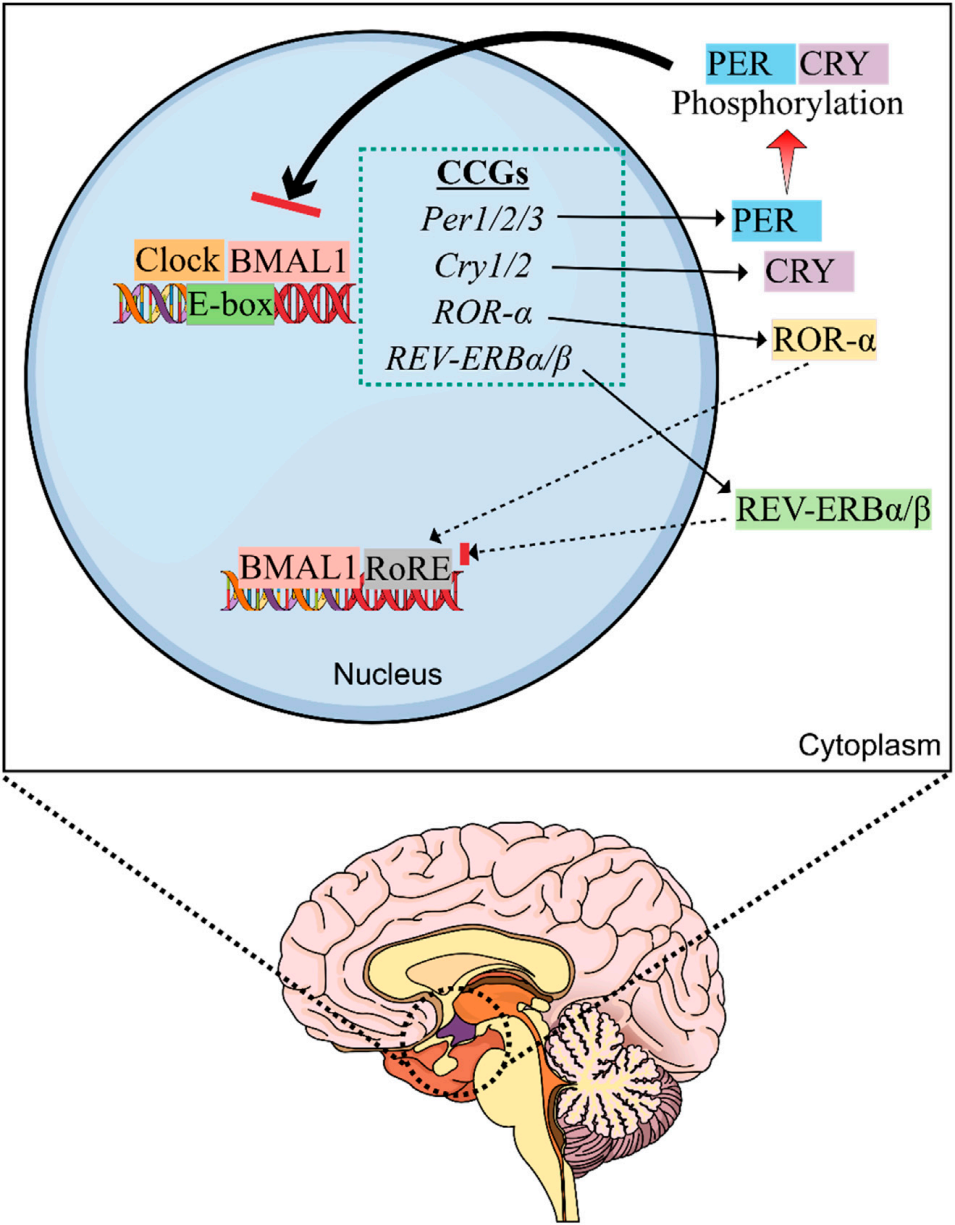
Molecular machinery of the circadian clock (CC). The master clock is located in the suprachiasmatic nucleus (SCN) of hypothalamus that controls the circadian rhythm. SCN is the central oscillator of the CC and controls most of the circadian rhythms in the body. Exposure to light provides the primary time cue for the CC present in SCN of the hypothalamus and inhibits the synthesis of melatonin by the pineal gland. Thus, exposure to artificial light at night can disturb SCN clock and melatonin rhythm. The heterodimeric CLOCK:BMAL1 complex is responsible for causing transcription of multiple circadian clock genes (CCGs) by interacting with the E-box elements on DNA. Following production, CRY and PER accrue in the cytoplasm and generate heterodimers that shuttle between the cytoplasm and nucleus. After being phosphorylated by CK1ε and CK1δ, they suppress the transcription of E-box genes via blocking CLOCK:BMAL1-induced transcription. REV-ERBα suppresses *Bmal1* expression via interacting with the ROR responsive element (RORE) in the promoter region, whereas RORα stimulates *Bmal1* expression.

Interestingly, when negative transcriptional feedback as well as post-translational and post-transcriptional regulation of CRY and PER is adequate to reduce the levels of PER/CRY proteins in the nucleus, suppression is relieved and CLOCK:BMAL1 mediates transcription of the *Per* and *Cry* genes (Takahashi, 2016a). A range of core mammalian CCGs, feedback loops, and various other genes have been discovered since the initial discovery (Figure 1). In the case of the second feedback loop, BMAL1 and CLOCK activate the transcription of genes for the nuclear receptors REV-ERBβ and REV-ERBα, which were found to compete with the retinoic acid-related orphan receptors (ROR-α, β, and γ) for ROR-binding sites on the *BMAL1* gene, which also provide both negative (REV-ERB) and positive (ROR) transcription regulation (Zhang et al., 2015). On the other hand, the third feedback loop includes the nuclear factor, interleukin 3 regulated (NFIL3) protein and D-box binding protein (DBP), which were found to be controlled by CRY1 and CLOCK:BMAL1, and bind with the D-box elements on the promoters of various CCGs including ROR-α and β (Stratmann et al., 2010). Collectively, CC is regulated by these feedback loops, which are controlled by transcriptional, post-transcriptional, and post-translational regulatory mechanisms that are adequate to maintain CCs (Golombek and Rosenstein, 2010a; Takahashi, 2016a; Cox and Takahashi, 2019).

## 4 Role of the circadian clock in the central nervous system

Oscillations of CC are seen in nearly all tissues (including glial cells), which play crucial roles in brain functions and development (Rojo et al., 2022). Since most of the malignancies in the brain initiate from glial cells or their precursors, therefore it is important to know the physiological functions of CC (Arafa and Emara, 2020). In the brain, astrocytes are the most plentiful population of glial cells (Allen and Eroglu, 2017). Glial cells are important for nervous system health because of their crucial trophic and metabolic support to neurons (Murphy-Royal et al., 2017). In addition, astrocytes showed rhythmic alterations governed by CCGs *in vitro* in mouse cortical astrocyte cultures. Oscillations of *Per1, Per2, Clock,* and IP3-dependent calcium signalling were found to control the daily rhythms of ATP secretion in astrocytes (Marpegan et al., 2011). The core CC protein BMAL1 regulates neurotrophic functions and astrocyte activation through a cell-autonomous mechanism, whereas reduced BMAL1 levels trigger astrogliosis (Lananna et al., 2018). The generation of hypoxic inducible factor 1α (HIF1α) was found to be induced in the brain by hypoxia in ependymal, astrocytes, neurons, and perhaps endothelial cells (Jolly et al., 2011a). It has been identified that ROR-α is a target for HIF1α (Jolly et al., 2011a). An increased level of ROR-α was detected *in vitro* during hypoxia in primary mouse astrocytes, which further resulted in the hypoxic inducible factor 1α downregulation (Jolly et al., 2011b). Glutamate uptake levels are influenced by *Npas2, Per2, Clock* along with no noticeable circadian variation. Interestingly, astrocytes in the SCN regulated behaviour and daily rhythms in the SCN, while *Bmal1* deletion in astrocytes extended the circadian period of rest-activity rhythms in mice (Tso et al., 2017a). Collectively, these findings indicate the crucial roles of CC in astrocyte functions (Quist et al., 2024).

Microglia are the major immune cells of the CNS that eradicate dead cells in adult CNS and the developing brain to mediate normal brain development (Prinz et al., 2019). They have a significant contribution in the development and preservation of synapses. In addition, microglia contain a CC that controls their immune activity (Fonken et al., 2015). Disturbances of CCGs in microglia can enhance chronic neuroinflammation which was found to be linked with the early onset of Alzheimer’s Disease (Ni et al., 2019). Obesogenic diets were found to affect the expression of CCG in microglia, which resulted in chronic microglial activation in rat models (Milanova et al., 2019). The molecular clock REV-ERBα plays an important role in establishing a balance in microglial phenotype and averting neuroinflammation. Synaptic phagocytosis increases because of the low REV-ERBα levels, whereas REV-ERBα loss can lead to neuronal dysfunction and spontaneous neuroinflammation (Griffin et al., 2019; 2020). Oligodendrocytes are neuroglial cells that have a significant contribution in signal conduction in the CNS (Kuhn et al., 2019). At present, there is no strong evidence that oligodendrocytes have an internal CC. On the other hand, rhythmic expressions of *Per2*, *Bmal1*, and *Rev-Erbα* have already been demonstrated in mice. Furthermore, *Bmal1* deletion showed a strong link between its expression and oligodendrocyte precursor cell-cycle regulation, cell proliferation, and morphology. Genes specific to oligodendrocytes oscillate throughout the sleep-wake cycle in mouse models. Thus, it is believed that oligodendrocytes might possess a functional CC. Since numerous studies have already demonstrated that CC regulates important events in functions of glial cells, thus disturbances in CC can lead to various neurological conditions (Quist et al., 2024).

## 5 Relationship between circadian clock genes and cancer

**FIGURE 2:**
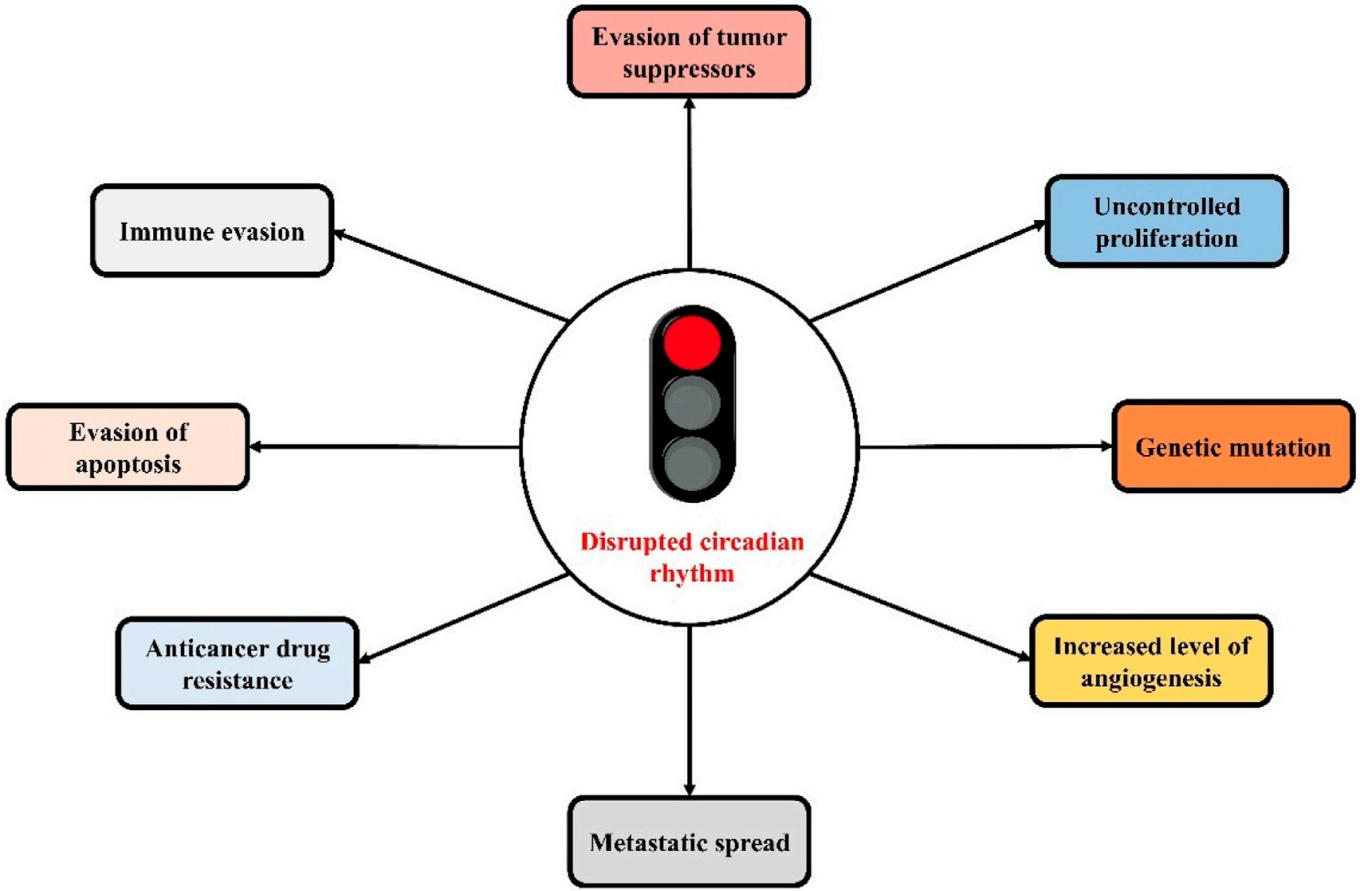
The close link between circadian clock disruptions and cancer hallmarks.

The close link between cancer and CC disruptions has already been demonstrated by various studies (Figure 2). Dysregulated expression of CCGs in various types of tumors has also been revealed (Shilts et al., 2018). There is a presence of a mutual regulatory process between CCGs and cancer genes. Several tumor suppressor genes and oncogenes (for example-tumor suppressor gene p53 and oncogene c-Myc) are regulated by CC, as well as the core CCGs are controlled by tumor suppressor genes and oncogenes, which are associated with malignancy and tumor onse (Huber et al., 2016; Shostak et al., 2016; Li, 2019). CC also controls gene rhythms linked with metabolic activity, endocrine functions, and metabolic functions as well as homeostasis of the endocrine system, which have a significant contribution in the development of tumors (Blask et al., 2014; Gamble et al., 2014; Aviram et al., 2016). Aberrant circadian rhythms mediate the malignant advancement of tumors via the deteriorating the immune system. As the immune system is crucial in limiting the development of tumors, therefore disturbed biological rhythms can affect the body’s innate and acquired immunity as well as mediate tumor immune escape via immune checkpoints (He et al., 2018; Wu et al., 2019a; Aiello et al., 2020; Hadadi et al., 2020).

SCN is an important bilateral structure located in the hypothalamus, which is the central pacemaker of the circadian timing system, which can receive (Duhart et al., 2017). The circadian cycles repeat with a period close to 24 h, even in the absenteeism of external stimulations, and it can adjust the alterations in the light-dark cycle via a certain synchronization cascade (Golombek and Rosenstein, 2010b). Various behavioral and physiological variables including neurological functions and hormone levels are controlled by the CC, which also have a significant contribution in the regulation of circadian rhythm (Musiek and Holtzman, 2016). In addition, misalignment and circadian disturbances between the CC and the environmental cycles have been linked with fatigue and mood disorders (Duhart et al., 2013b; Bedrosian and Nelson, 2017). Within the SCN, glial cells are associated with synchronization mechanisms (Lavialle et al., 2001; Girardet et al., 2010; Duhart et al., 2013b) and circadian timekeeping (Barca-Mayo et al., 2017; Brancaccio et al., 2017; Tso et al., 2017b), and are also regarded as mediators between the circadian pacemaker and proinflammatory signals (Duhart et al., 2013a). Among the proposed mechanisms for glial cells-mediated modulation of the circadian pacemaker, the modulation of glutamate concentrations via SCN astrocytes is crucial for the appropriate CC functioning (Leone et al., 2015; Brancaccio et al., 2017).

Elevated levels of glutamate are characteristic features of gliomas, which indicates that a disequilibrium in proper glial cell functions takes place in malignant tissues, which might affect both synchronization and timekeeping mechanisms of the clock (Robert and Sontheimer, 2013). Moreover, various molecules associated with immune responses including CCL2, IL-1β, and TNF-α can influence the master circadian oscillator (Leone et al., 2012; Duhart et al., 2016). Gliomas can markedly change the TME in which they develop, and molecules associated with the immune responses have been confirmed to have a significant contribution in the progression of tumors (Christofides et al., 2015). In view of the occurrence of fatigue and sleep alterations as common symptoms of gliomas, the disruption in anatomical features of CC and optic tract in hypothalamic gliomas, and the changes in the molecules pertinent for the circadian pacemaker in TME (Duhart et al., 2017).

## 6 The link between pathological disruptions of the circadian clock genes and brain tumor

### 6.1 Bmal1 gene

*Bmal1* is the only gene among the CCGs whose elimination can eradicate circadian rhythmicity. *Bmal1* is crucial in cell-autonomous transcription-translation feedback loops. Modification of CC because of mutations in *Bmal1* can either induce the growth of tumors or the entire carcinogenesis process. Various studies have detected mutated or dysregulated core CCGs including *Clock* and *Bmal1* in cancer cells (Ye et al., 2018; Battaglin et al., 2021; Qu et al., 2023). In addition, disrupted or mutated *Bmal1* contributes in all stages of cancer including tumorigenesis, protein synthesis, tumor progression, as well as metastasis (Huang et al., 2023). In a study, Zeng et al. assessed the *in vitro* and *in vivo* outcome of *Bmal1* knockdown by RNA interference. They observed that *Bmal1* downregulation can induce tumour development, which can further affect the response to anti-cancer drugs (Zeng et al., 2010). This takes place because of the growth proliferation rate following CC disturbance as tumor suppressors and important cell cycle genes are regulated by CC (Table 1). Disrupted CC is a common characteristic of advanced-stage tumors and cancer cell lines (Relógio et al., 2014). Knockdown of *Bmal1* in B16 tumors averted the actions of dexamethasone on cell cycle events and tumor growth (Kiessling et al., 2017). In addition, both increases and decreases in *Bmal1* expression are linked with glioma biology. Overexpression of *Bmal1* in patients with high-grade glioma-mediated suppression of GBM cell growth (Casili et al., 2022).

**TABLE 1:** A summary of the roles of disrupted circadian clock genes in brain tumors.

Circadian clock genes	Molecular mechanisms of disrupted circadian clock genes in brain tumor	References
*Bmal1*	*Bmal1* plays an important role in regulating microglial immune function and cellular metabolism. The CLOCK:BMAL1 triggers microglial reprogramming through the HIF1α/OLFML3/LGMN axis. This microglial reprogramming can inhibit immune cells, mediate tumorigenesis and obtain an infiltrative phenotype. In addition to this, reprogrammed microglia signal back to the tumor cell via exosomes with miR-7239-3p to reduce *Bmal1* and elevate *Clock*	Wang et al. (2020), Munteanu et al. (2024)
*Clock*	After CC suppression, a decreased level of proliferation and apoptosis induction was detected in gliomas associated with a p53 complex upregulation, which indicates the anti-apoptotic modulation of *Clock* in gliomas. An opposite link was observed between the tumor-suppressive action of the *Clock* and hypoxia, which indicates that the *Clock* suppression in tumor cells increased survival and decreased microglial migration	De La Cruz Minyety et al. (2021), Xuan et al. (2022)
*Period 1*	A lower-level expression of *Per1* was detected in high-grade glioma cell lines in comparison with the adjacent non-tumor-bearing tissues. In addition, deregulation in the expression of *Per1* facilitated gliomas in proliferation and survival, as this was linked with a disturbance of the clock activity	Xia et al. (2010)
*Period 2*	The lower level of *Per2* expression was found to be linked with high-grade gliomas and elevated expressions of epidermal growth factor receptors as well as proliferating cell nuclear antigens	Wang et al. (2018)
*Period 3*	The expression level of *Per3* is reduced in the case of gliomas, which is linked with higher mortality	Madden et al. (2014), Chang and Lai (2019)
*Cry1*	Increased levels of *Cry1* in patients with GBM as compared to normal brains	Wang et al. (2021)
*Cry2*	A reduced level of *Cry2* expression was observed in gliomas than in healthy tissues and linked with an increased mortality rate	Luo et al. (2012), Chang and Lai (2019), Wang et al. (2021)
*ROR-α*	A lower level of *ROR-α* expression has been observed in gliomas and the overexpression of *ROR-α* is linked with higher survival rates	Jiang et al. (2020)
*REV-ERBα* and *REV-ERBβ*	Both REV-ERBα as well as REV-ERBβ have significant contributions in circadian rhythms, inflammation, tumorigenesis, and glucose as well as lipid metabolism, which play a role as the components associating the CC with the cellular metabolism. An elevated level of *NR1D2* (*REV-ERBβ*) expression was found to correlate positively with glioma grades. Compared to healthy noncancerous astrocytes, increased levels of *NR1D2* were found in glioblastoma multiforme cells	Yu et al. (2018), Chan et al. (2023)

### 6.2 Clock gene

An important CCG is the *Clock gene, which is* located at the 4q12 chromosomal region, which encodes a transcription factor that is supposed to influence both the period and persistence of CCs. The *Clock gene acts as an* activator of downstream elements in the signalling pathway which is important for the generation of CCs (Bacchelli et al., 2016; Burish et al., 2018; 2019). Sleep duration was found to be linked with the 2 single-nucleotide polymorphisms including rs11932595 and rs12649507 found in the intronic area of the *Clock* gene. A circadian behavior has been observed in BTs and the CC disruption is linked with a higher occurrence of tumors (Janich et al., 2011). The *Clock gene can act as a*n oncogene in some tissues to promote cancer, while in some other tissue it plays a role as an inhibitor of tumors (Fekry et al., 2018). Both *BMAL1* and *Clock* genes can play roles as oncogenes in case of GBM. These genes are crucial for the proliferation and survival of GSCs, however they are not essential for differentiated GBM cells or normal neural stem cells (García-Costela et al., 2020). Neuronal cancer cells have an increased demand for nutrients because of their rapid growth, which can shift their metabolism toward the usage of glycolysis instead of oxidative phosphorylation in order to produce ATP; therefore several components of the metabolic pathway are changed in tumors (Lunt and Vander Heiden, 2011; Casili et al., 2022). Disturbances in CC through mutations in the circadian pathway or by environmental factors can result in a higher risk of tumorigenesis (Marin-Valencia et al., 2012; Agnihotri and Zadeh, 2016; Sulli et al., 2019). In the case of cancer, inconsistencies in the *Clock* gene expression can trigger modifications in the activations and/or inhibitions of the main tumor suppressive and oncogenic signalling pathways (Patel et al., 2014). Indeed, knowledge regarding the *Clock-associated processes in tumors can prove beneficial in the development of* tumor therapy. Moreover, transforming growth factor-β plays the role as a crucial CC regulator, which controls the expression of both negative and positive switches of CC oscillations (Gao et al., 2021).

### 6.3 Period 1 gene

The *Per1* gene is the master regulator of circadian rhythms which is responsible for encoding PER1 protein. A lower-level expression of *Per1* was detected in high-grade glioma cell lines than in adjacent non-tumor-bearing tissues. In addition, deregulation in the expression of *Per1* facilitated gliomas in proliferation and survival, as this was linked with a disturbance of the clock activity (Xia et al., 2010). A decreased level of *Per1* expression has also been reported in high-grade gliomas (Wang et al., 2021). Furthermore, tumors developed by injection of cells derived from a malignant peripheral nerve sheath tumor showed lower levels of Per1 mRNA as compared to normal tissues (Wagner et al., 2021b). In a study, researchers observed *Per1* overexpression and detected a variant of *Per1*, rs2289591, which was found to be linked with glioma risk and mortality in patients with high-grade glioma (Madden et al., 2014). Expression of *Per1* was linked with the radiosensitivity of gliomas in culture; the downregulation of *Per1* weakened the radiosensitivity of U343 glioma cell lines, which resulted in reduced apoptosis of irradiated tumor cells. Knockdown of *Per1* reduced the levels of p53 and CHK2 proteins, critical DNA damage checkpoints. It has been proposed that *Per1* as a tumor suppressor gene controls the p53 pathway and affects the levels of p53 with a direct effect on proliferation suppression and apoptosis promotion (Zhu et al., 2019). In a similar manner, increased *Per1* expression is linked with elevated radiosensitivity in gliomas in rats, whereas this finding was not confirmed in non-tumor tissues. The levels of *Per1* exhibit circadian patterns of gene expression in both tumor and normal tissues. Normal tissues showed around 24 h periodicity on *Per1* expression, while gliomas showed a 12-h periodicity. It was also observed that *Per1* exhibits a tumor suppressive role in gliomas and the expression of *Per1* is linked with increased x-ray sensitivity and cell cycle arrest (Zhanfeng et al., 2015). Mutation in the isocitrate dehydrogenase 1 (*IDH1*) gene (R132H) was found to be linked with a decreased level of GBM cell proliferation and altered levels of CCGs, along with a reduced *Per1* expression (Gao et al., 2021). Collectively, these findings indicate that tumor cells might show abnormal oscillations in *Per1* expression, which can eventually affect tumor survival and cell proliferation (Wagner et al., 2021a).

### 6.4 Period 2 gene

A disrupted level of *Per2* expression has been observed in gliomas as compared to normal brain tissues. It has also been observed that there is a lower level of *Per2* expression in non-glioma cells, which indicates the differences in CCG expressions between malignant and normal brain tissues (Xia et al., 2010). The lower level of *Per2* expression was found to be linked with high-grade gliomas and elevated expressions of epidermal growth factor receptors as well as proliferating cell nuclear antigens (Wang et al., 2018). In a study, it was observed that disruption in the cell signalling pathway or promoter methylation might affect the expression of *Per2* in tumor tissues (Wang et al., 2018). Similarly, the downregulation of *Per2* was observed in samples derived from The Cancer Genome Atlas (TCGA) database (Chen et al., 2013), and *Per2* deregulation in tumors was found to be linked with increased mortality in the cohort of patients with glioma (Chang and Lai, 2019). The critical role of *Per2* has also been revealed in the case of gliomagenesis. The levels of *Per2* mRNA and protein were found to be downregulated in GSCs, and their overexpression impaired its proliferation via the cell cycle, arresting them in G_0_/G_1_ phase. As *Per2* can target the Wnt/β-catenin signalling cascade in GSCs, the downregulation of important proteins associated with the stemness and invasiveness of GSCs, including c-Myc, MMP9, MMP2, β-catenin, and Wnt7b, might explain the tumor suppressive properties of *Per2* in gliomas (Ma et al., 2020; Hakami et al., 2024b). Furthermore, the R132H mutation in the *IDH1* gene was found to be linked with reduced protein levels for *Per2* (Gao et al., 2021; Wagner et al., 2021a).

### 6.5 Period 3 gene

The expression level of *Per3* is reduced in the case of gliomas, which is linked with higher mortality (Madden et al., 2014; Chang and Lai, 2019). Studies involving TCGA samples showed a decreased *Per3* level of expression in GBM samples (Wang et al., 2021). The R132H mutation in the *IDH1* gene is also linked with a decreased level of *Per3* expression (Gao et al., 2021). Collectively, these findings indicate the significance of the *Per3* gene as a tumor suppressor in gliomagenesis. However, more studies are required to investigate the activity of *Per3* gliomagenesis (Wagner et al., 2021a).

### 6.6 Cryptochrome genes

Both PER and CRY proteins are important for the preservation of cellular circadian homeostasis. In a study, a downregulated *Cry1* expression in gliomas was observed in 69 patient samples as compared to non-tumor cells (Luo et al., 2012). In contrast, an analysis of the TCGA database observed increased *Cry1* levels in patients with GBM as compared to normal brains (Madden et al., 2014; Wang et al., 2021). The *IDH1* gene mutations were markedly associated with a downregulated expression of *Cry1* in U-87 MG cell lines in comparison with the control cells. Mutations in *IDH1* gene influence glioma proliferation via the TGF-ß/Smad signalling cascade by modifying CCG expression (Gao et al., 2021). The contributions of the *Cry1* gene in glioma biology have been demonstrated in experimental models of *Cry1/2* double knockout mouse models under chronic jetlag conditions. Collectively, these findings indicate a relationship between CCGs and glioma-associated genes as well as the effect of light conditions in carcinogenesis (Khan et al., 2019). A reduced level of *Cry2* expression was observed in gliomas than the healthy tissues and linked with an increased mortality rate (Luo et al., 2012; Chang and Lai, 2019; Wang et al., 2021). Nonetheless, in a rat model, results obtained from irradiated gliomas indicated a link between elevated *Cry2* expression and elevated cell proliferation as well as reduced apoptosis. However, more studies are required to demonstrate the effects of *Cry2* in the development of GBM (Wagner et al., 2021a).

### 6.7 *ROR-α* gene

A lower level of *ROR-α* expression has been observed in gliomas and the overexpression of *ROR-α* is linked with higher survival rates in Chinese Glioma Genome Atlas and TCGA data. The lower levels of *ROR-α* are linked with poor prognosis in the case of GBM. As compared to healthy tissues, *ROR-α* level is markedly lowered in grade II to IV gliomas. On the other hand, *ROR-α* overexpression reduced the extent of cell proliferation as well as triggered cycle arrest in T98G cells and GSC4D GBM cell lines as well as GSCs and suppressed *in vivo* tumorigenesis. Gene set enrichment analysis (GSEA) showed that low levels of *ROR-α* expressions were linked with the TNF-induced signalling cascade, and glioma samples showed a negative relationship between *TNF*-α as well as *ROR-α*. In addition, *ROR-α*-induced TNF-α suppression resulted in downstream suppression of nuclear factor kappa B (NF-κB) signalling cascade, which plays a role in antiproliferative activities of *ROR-α* in glioma. It has been observed that miR-18a can negatively control *the* expression of *ROR-α* via binding with its 3ʹ-UTR, and mrR-18a also can cause NF-κB and TNF-α signalling cascade activations (Jiang et al., 2020). Nonetheless, more studies are required to elucidate the roles of *ROR-α* in GBM (Chan et al., 2023).

### 6.8 REV-ERBα and REV-ERBβ genes

The nuclear receptors REV-ERBα and REV-ERBβ are encoded by the *NR1D1* (Nuclear Receptor Subfamily 1 Group D Member 1) and *NR1D2* (Nuclear Receptor Subfamily 1 Group D Member 2) genes, respectively. These nuclear receptors have significant contributions in circadian rhythms, inflammation, tumorigenesis, and glucose as well as lipid metabolism, which play a role as the components associating the CC with the cellular metabolism (Wagner et al., 2021a). An elevated level of *NR1D2* (*REV-ERBβ*) expression was found to correlate positively with glioma grades. Compared to healthy noncancerous astrocytes, increased levels of *NR1D2* were found in GBM cell lines. In addition, *NR1D2* depletion through siRNA reduced cell viability, invasion, and migration, as well as elevated G1-phase populations in GBM cells than human astrocytes. FA (focal adhesion) and EMT were detected as *REV-ERBβ* target genes, therefore *NR1D2 (REV-ERBβ)* might serve as a potential target for GBM treatment via suppressing the invasion and migration of GBM cells (Yu et al., 2018; Chan et al., 2023). Overall, aberrant expression of CCGs influences tumour prognosis via affecting tumour cell proliferation and tumour immune landscape (Wang et al., 2021).

## 7 Therapeutic interventions to modulate circadian clock genes in brain tumors

### 7.1 Temozolomide

TMZ is an alkylating agent and an anticancer medication, which exerts its action via DNA methylation at the O^6^-guanine residue site (Zhang et al., 2012). TMZ triggers DNA cross-linking and ultimately cell apoptosis through DNA methylation (Zhang et al., 2012). O^6^-methylguanine-DNA methyltransferase (MGMT) enzyme plays a role in repairing DNAs, which has a significant contribution in chemoresistance to TMZ. However, a subset of GBM showed that methylation of MGMT causes inactivation of the repair enzyme and results in prolonged patient survival due to tumor cell TMZ sensitivity (Zhang et al., 2012). In adults, gliomas showed differential responses according to the time of administration of TMZ, which indicates a useful role for TMZ chronotherapy (Damato et al., 2021; Chai et al., 2022). In a study with adult GBM patients, it was reported that TMZ administration in the morning resulted in 3.6 months longer overall survival than patients who administered TMZ in the evenings (Damato et al., 2021). Furthermore, TMZ administration in the morning extended this survival to a 6-month elevated overall survival in MGMT-methylated GBM patients compared to the MGMT-methylated GBM patients who received TMZ in the evening (Damato et al., 2021). Based on these results, a follow-up phase II clinical trial was carried out, which revealed that TMZ chronotherapy is feasible (Damato et al., 2022).

Even though this study did not find any difference in adverse effects or overall survival between individuals administered TMZ in the evening versus morning, however the researchers concluded that the heterogenous patient population and the small sample size limit the inference of the study regarding survival benefit (Damato et al., 2022). In general, elevated sensitivity exhibited by TMZ in the morning might be induced by differential and diurnal expressions of MGMT (Nettnin et al., 2023). It is also believed that the direct interaction of TMZ with CCG expression is linked with the differential TMZ efficacy in gliomas. Supposedly, the sensitivity of TMZ is particularly linked with the cyclic expression of the *Bmal1* gene (Takahashi, 2016b). Researchers observed that when TMZ was administered close to the daily peak in *Bmal1* expression, both primary mesenchymal murine GBM astrocytes and primary human GBM were most sensitive to TMZ (Slat et al., 2017). Deletion of *Bmal1* by clustered regularly interspaced short palindromic repeats (CRISPR) diminished the aforesaid temporal effect, which indicates that the sensitivity of TMZ chronotherapy is reliant on *Bmal1* (Slat et al., 2017). In a different study, bioinformatics analyses confirmed that increased *Bmal1* expression is greatly linked with enhanced TMZ sensitivity (Chai et al., 2022). Collectively, these results indicate the relationship between the molecular components of CC and the outcomes of TMZ chronotherapy. In order to ensure optimum benefit, more studies along with randomized control trials are required to determine the best time for TMZ administration (Jia et al., 2023).

### 7.2 Inhibitors of casein kinase 1 (CK1) and casein kinase 2 (CK2)

CK1 and CK2 inhibitors are potential candidates for averting the degradation of PER1/2, which might be effective in potentiating the anti-tumor activities of the period (PER) proteins in the case of GBM. Treatments with CK1 inhibitors including PF-670462 and longdaysin were found to affect CCs perhaps by suppressing CK1-induced PER phosphorylation and its successive degradations (Knippschild et al., 2014). Longdaysin suppressed the Wnt/β-Catenin signalling cascade and reduced sphere formation, invasion, migration, and colony generation of breast cancer cells. In addition, longdaysin averted the growths of triple-negative breast cancer xenografts *in vivo* (Xiong et al., 2019). On the other hand, PF-670462 prevented the interactions between TME and chronic lymphocytic leukemia cell lines, which resulted in reduced *in vivo* progression as well as delayed chronic lymphocytic leukemia onset and increased overall survival (Janovska et al., 2018). GO289 (a specific CK2 inhibitor) was found to modulate CCs and avert phosphorylation as well as degradation of PERs. As GO289 shows specificity towards CK2, therefore it does not bind with the hinge area that is highly conserved across kinases. Moreover, GO289 decreased the growth of various mouse MLL-AF9 acute myeloid leukemia and renal cell carcinoma cell lines (Oshima et al., 2019; Chan et al., 2023).

### 7.3 Curcumin

Curcumin, a polyphenolic phytochemical, can be used in the treatment of glioma (Xiong et al., 2021). Curcumin administration results in the alteration of molecular circadian timing within cells. It has the capacity to modulate the expressions of *NF-kB*, *PPAR-γ*, and *STAT* within two interacted molecular timing loops (Sarma et al., 2016; Xiong et al., 2021). It has been reported that curcumin can suppress the NF-κB-dependent signalling pathway, which can lead to tumor shrinkage and apoptosis induction (Islam et al., 2024). Moreover, curcumin was found to arrest GBM stem cells via suppressing both STAT3 and the inhibitor of apoptosis-dependent signalling pathway, along with activation of the MAPK signalling cascade. This phytochemical also activates *Bmal1* through PPAR-γ induction. Curcumin causes activation of sirtuin 1, subsequently the activated binds with heterodimeric CLOCK:BMAL1 in order to mediate the deacetylation and PER2 degradation. Curcumin treatment at the dose of 10 μM disrupted a single circadian oscillator within the CC unit or the coupling between CCs in apoptosis (Damato et al., 2022). Following the determination of the circadian phase, curcumin or its analogs as an anticancer therapy ought to be administered to tumor cells at the optimum stage to maximise effectiveness (Wang and Chen, 2022).

### 7.4 Norepinephrine

Norepinephrine mainly acts in the brain stem, which is associated with various behaviors including awakening and sleep (Wang X. et al., 2022). Norepinephrine also plays a role in the pinealocytes of the pineal gland. It acts through cAMP to activate arylalkylamine N-acetyltransferase, the key enzyme needed for melatonin biosynthesis (Wang X. et al., 2022). Administration of norepinephrine increased the expression of *Per1* mRNA through β2-adrenergic receptors (Wagner et al., 2019a). Moreover, this same area may be associated with the actions of both the Src family of protein tyrosine kinase and protein kinase A. The protein kinase A-cAMP-response element binding protein signalling pathway coupled with β2-adrenoceptors has a significant contribution in controlling CCGs including *Per1* in chondrocytes and cerebellar granule cells (Wang and Chen, 2022; Wang X. et al., 2022).

### 7.5 Melatonin

A number of studies have already confirmed the link between CCG disruption, cancer and reduced levels of melatonin. In addition, inhibition of melatonin generation is linked with an elevated occurrence of cancer (Rodríguez-Santana et al., 2023). Light is crucial in synchronizing cellular homeostasis as well as circadian machinery and controls the levels of melatonin. Melatonin is a natural hormone synthesized by the pineal gland, which regulates CC by regulating sleep and wake cycles. Treatment with various melatonin agonists is extensively utilized to treat neuropsychiatric disorders, synchronize CCs, and control sleep disruptions (Satyanarayanan et al., 2018). It has been observed that melatonin also exerts antiproliferative properties and suppresses the growth of various tumour types (Maitra et al., 2019). In a study, melatonin showed *in vivo* suppressive action on the proliferation of neural stem cells found in the subventricular zone (Gengatharan et al., 2021). Furthermore, reduced levels of melatonin synthesis and secretion of its receptors in the subventricular zone mediate the initiation and growth of GBM (Ghareghani et al., 2022). Melatonin might exert a therapeutic action by decreasing the proliferation of GBM cells and disturbance of light-dependent melatonin synthesis, which indicates the link between GBM and melatonin. Melatonin also decreases chemotherapeutic drug resistance in GBM stem cells (Chen et al., 2016; Guerrero-Vargas et al., 2017). Collectively, these findings indicate the suppressive action of melatonin in GBM (Petković et al., 2023).

### 7.6 Agonists of REV-ERB

REV-ERBs play an important role in CC. Agonists of REV-ERBs including SR9011 and SR9009 are particularly lethal to oncogene-induced senescent cells and cancerous cells, however they do not influence the viability of normal tissues or cells (Sulli et al., 2018). Treatment with SR9009 elevated the lipid droplet levels and reduced the levels of reactive oxygen species, while the combination of bortezomib and SR9009 exerted synergistic or additive effects in T98G cells (Sulli et al., 2018). In malignant cells, treatment with REV-ERB agonists and autophagy regulation showed effectiveness in inducing apoptosis (Wagner et al., 2019a). Moreover, REV-ERB agonists impaired GBM growth *in vivo,* exerted selective anticancer effects, and ameliorated survival along with no overt toxic effects in mouse models (Wagner et al., 2019a). The agonists of REV-ERBs can affect various oncogenic drivers (such as-PIK3CA, BRAF, and HRAS) and can also continue in TP53 absence and under various hypoxic conditions (Wang and Chen, 2022).

### 7.7 ROR agonists

The compounds that target ROR have been assessed in GBM, however use of these agents to suppress immunosuppression might be beneficial as they can act as anti-tumorigenic via modifying the GBM TME. SR1078 is an agonist of RORα/γ, which inhibited NF-κB function and enhanced CD8^+^ T-cell responses in the Jurkat T cell leukemia cell line (Lee et al., 2020). Agonists of RORγ including LYC-54143 and LYC-53772 blocked differentiation of Th17 cells as well as immunosuppression and also elevated cytokine levels, thus showed anti-tumor properties by both reducing immune suppression and enhancing immune activation. LYC-54143 also increased the cytotoxic effect of Tc17 cells and enhanced *in vitro* CAR-T cell-induced targeting of K562 cancer cells whereas also suppressing *in vivo* 4T1 breast tumor and MC38 colorectal growth (Hu et al., 2016).

### 7.8 Cryptochrome protein (CRY) stabilizers

**FIGURE 3:**
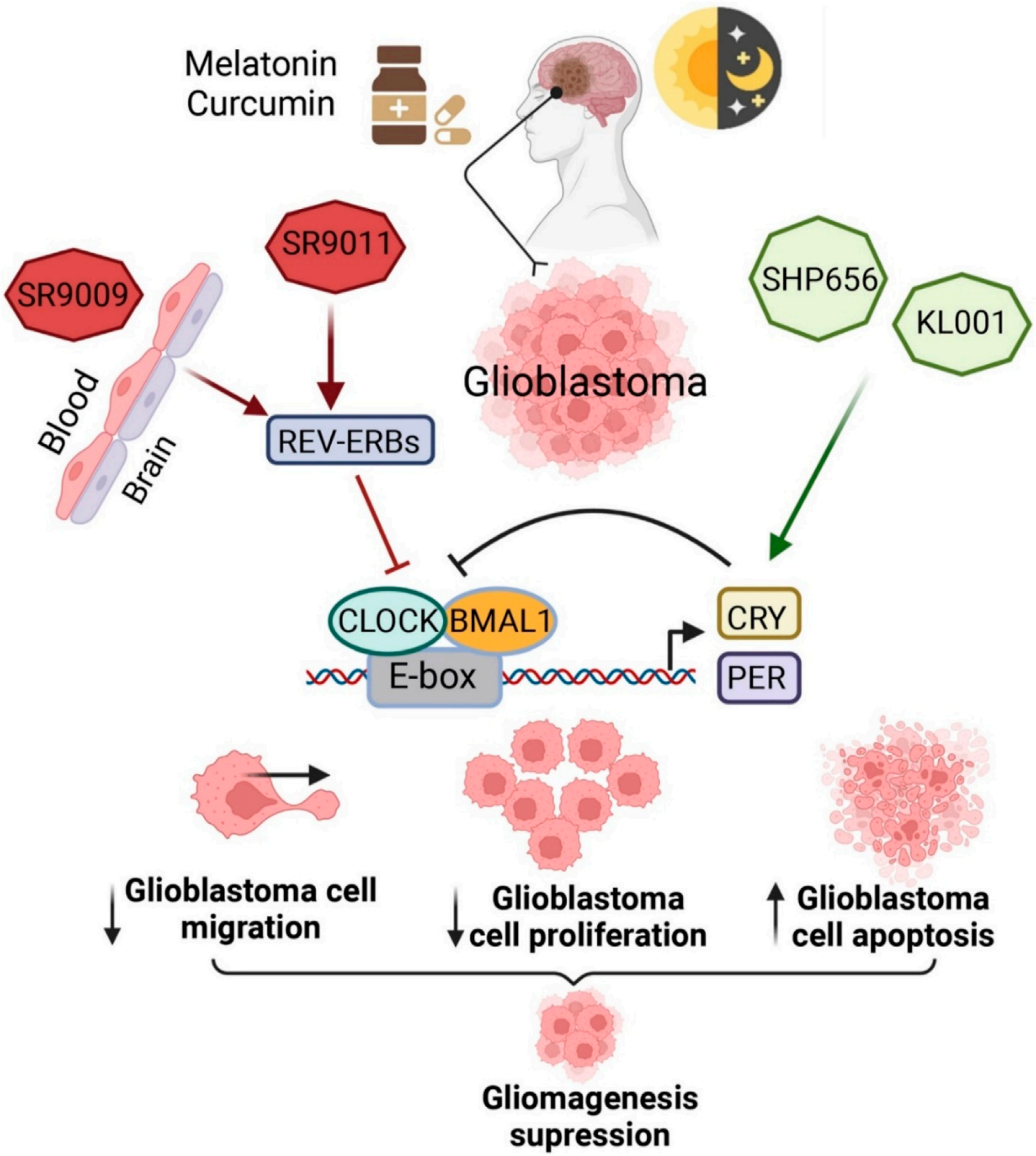
Pharmacological interventions to modulate circadian clock genes in BTs. This figure is adapted from (Petković et al., 2023), used under a CC BY license.

Treatment with the first CRY stabilizer, KL001, increased the length of the period and decreased amplitude in a dose-dependent manner in U2OS cells containing a *Per2-dLuc* or *Bmal1-dLuc* reporter. KL001 has the capacity to interact with both the CRYs (Figure 3) and stabilize the proteins through suppressing FBXL3-induced compound degradations by interacting with their flavin adenine dinucleotide binding pocket (Hirota et al., 2012). Interestingly, KL001 reduced migration, survival, stemness, self-renewal, and CCG expressions in GSCs as compared to healthy or differentiated GBM cells. Treatment with the combination of REV-ERB agonists (SR9011 and SR9009) and KL001 resulted in a synergistic effect, which indicates that targeting both negative limbs of the circadian transcription-translation feedback loop can result in enhanced GSC targeting. A different CRY stabilizer, SHP656, was developed according to KL001 to enhance brain penetration and bioavailability, which substantially decreased the proliferation of GSCs than noncancerous or differentiated GBM cells. SHP656 also extended the survival of mouse models with tumors induced from two different patient-derived GSCs, which indicates the efficacy of CRY stabilizers in GBM treatment (Dong Q. et al., 2019). Various CRY stabilizing compounds have also been developed that are specific to isoforms (Miller et al., 2020; Kolarski et al., 2021). Use of these compounds might provide therapeutic advantages in case of the aforesaid variations in expressions of CRY1 or CRY2 in GBM tissues (Li et al., 2013).

### 7.9 1A-116

The 1A-116 compound is a Rac Family Small GTPase 1 (RAC1) inhibitor, which has been derived from its parental compound ZINC69391. It was reported that 1A-116 has the capacity to treat GBM and various other tumors (González et al., 2020; Sauzeau et al., 2022). In addition, in U-87 and LN229 glioma cells, 1A-116 selectively suppressed activation of RAC1 to guanine exchange factors including T-lymphoma invasion and metastasis-inducing protein 1 via interacting with the Trp56 residue (Cardama et al., 2014; Langston et al., 2019). Optimized time for 1A-116 administration can further optimize its efficacy. Enhanced 1A-116 efficacy was reported at low-level expression of *Bmal1* in GBM cells and a differential overall survival was observed when applying 1A-116 at Zeitgeber times 12 (ZT 12) to nude mouse models with gliomas (Trebucq et al., 2021). Indeed, the time-dependent administration of 1A–116 can enhance overall survival (Wang and Chen, 2022) (Table 2).

**TABLE 2:** An outline of drugs that can be used to modulate circadian clock genes in brain tumors.

Drugs	Mechanism of action	References
Temozolomide	In adults, gliomas showed differential responses according to the time of administration of temozolomide, which indicates a useful role for temozolomide chronotherapy. In a study with adult glioblastoma multiforme (GBM) patients, it was reported that temozolomide administration in the morning resulted in 3.6 months longer overall survival than patients who received temozolomide in the evenings	Damato et al. (2021), Chai et al. (2022)
Casein (CK1 and CK2) inhibitors	CK1 and CK2 inhibitors are potential candidates for averting the degradation of PER1/2, which might be effective in potentiating the anti-tumor activities of period (PER) proteins in the case of GBM.	Knippschild et al. (2014)
Curcumin	Curcumin activates *Bmal1* through PPAR-γ induction. In addition, it causes activation of sirtuin 1, subsequently the activated sirtuin 1 binds with heterodimeric CLOCK: BMAL1 in order to mediate the deacetylation and PER2 degradation	Sarma et al. (2016), Xiong et al. (2021)
Norepinephrine	Norepinephrine acts through cAMP to activate arylalkylamine N-acetyltransferase. Administration of norepinephrine increased the expression of *Per1* mRNA through β2-adrenergic receptors	Wang and Chen (2022), Wang et al. (2022b)
Melatonin	Melatonin decreases the proliferation of GBM cells and the disturbance of light-dependent melatonin synthesis, which indicates the link between GBM and melatonin. Melatonin also decreases chemotherapeutic drug resistance in GBM stem cells	Chen et al. (2016), Guerrero-Vargas et al. (2017)
Agonists of REV-ERB	In malignant cells, treatment with REV-ERB agonists and autophagy regulation showed effectiveness in inducing apoptosis. Moreover, REV-ERB agonists impaired GBM growth *in vivo,* exerted selective anticancer effects, and ameliorated survival along with no overt toxic effects in mouse models. The agonists of REV-ERBs can affect various oncogenic drivers (such as- PIK3CA, BRAF, and HRAS) and can also continue in TP53 absence and under various hypoxic conditions	Wagner et al. (2019a)
ROR agonists	SR1078 is an agonist of RORα/γ, which inhibited NF-κB function and enhanced CD8^+^ T-cell responses in the Jurkat T cell leukemia cell line. Agonists of RORγ including LYC-54143 and LYC-53772 blocked differentiation of Th17 cells as well as immunosuppression and also elevated cytokine levels, thus showed anti-tumor properties by both reducing immune suppression and enhancing immune activation	Hu et al. (2016), Lee et al. (2020)
Cryptochrome protein (CRY) stabilizers	CRY stabilizer, KL001, reduced migration, survival, stemness, self-renewal, and CCG expressions in GSCs as compared to healthy or differentiated GBM cells	Dong et al. (2019b)
1A-116	1A-116 selectively suppressed activation of Rac Family Small GTPase 1 in U-87 and LN229 glioma cells	Cardama et al. (2014), Langston et al. (2019)

## 8 Challenges and future prospects

Among the BTs, GBM is the most complex, aggressive, and treatment-resistant cancer. Despite global efforts, still insignificant improvement has been achieved in the improvement of overall survival of patients with BTs, particularly in the case of GBM. Growing evidence regarding gliomas including GBM indicates the significance of modulation of CCG in cancer biology. These studies also revealed how tumor cells can disrupt CCGs to safeguard their survival. It has also recently been demonstrated in the case of gliomas (especially GBM) that CCGs should be targeted for the development of novel therapies or to ameliorate the current treatments that impair and abolish tumor growth. Circadian rhythms present in cells are considered in the case of chronotherapy in order to estimate the optimum time for drug administration to enhance the therapeutic outcome as well as reduce undesirable side effects. However, still more studies are still required to answer several questions in this field including whether the circadian rhythm alteration is a crucial physiological factor in the genesis of glioma, whether the regimen of treatment is modified as per the CCG of the patient, and how to precisely detect CCGs of patients during clinical therapies. The presence of BBB is a major challenge in BT treatment, since BBB is composed of various transport systems and molecular components which can hinder the entry of various drugs in brain (Bhowmik et al., 2015; Arvanitis et al., 2019). Thus, this challenge also need to be carefully considered while developing novel therapies to treat BT.

## 9 Conclusion

Recent study findings have revealed that how tumor cells can reprogram CC to ensure their survival, which indicate the significance of CC modulation in case of cancer biology. There is a growing need for the targeting of CCGs to prevent and/or treat diseases particularly BTs, owing to the recent advances in CCG research and findings regarding the link between the CCG and molecular mechanisms controlling various physiological and pathological mechanisms. As mentioned above, dysregulation of CCGs is closely connected with the onset and progression of BTs. Since BTs including GBM show resistance to typical treatments with a bad prognosis, thus circadian consideration might prove beneficial in the case of treatments. In spite of significant findings revealed by various *in vitro* and animal studies, a low number of clinical studies have been carried out in this field. Therefore, more clinical trials are required to determine the optimum administration methods of drugs discussed above. More studies are also required to answer several questions in this field, such as how to precisely detect the patient’s CC status during clinical treatment and whether a customized treatment regimen is required based on the CC of the patient.
